# RNAi-mediated knockdown of *E2F2* inhibits tumorigenicity of human glioblastoma cells

**DOI:** 10.3892/ol.2014.2369

**Published:** 2014-07-22

**Authors:** ADRIANA M. NAKAHATA, DANIELA E. SUZUKI, CAROLINA O. RODINI, MAYARA L. FIUZA, OSWALDO K. OKAMOTO

**Affiliations:** 1 Department of Neurology and Neurosurgery, Experimental Neurology Unit, Federal University of São Paulo, São Paulo 04023-900, Brazil; 2 Department of Genetics and Evolutionary Biology, Human Genome and Stem Cell Research Center, Institute of Biosciences, University of São Paulo, São Paulo 05508-090, Brazil

**Keywords:** E2F2, glioblastoma multiforme, cancer, tumorigenesis

## Abstract

In a previous genome-wide expression profiling study, we identified *E2F2* as a hyperexpressed gene in stem-like cells of distinct glioblastoma multiforme (GBM) specimens. Since the encoded E2F2 transcription factor has been implicated in both tumor suppression and tumor development, we conducted a functional study to investigate the pertinence of *E2F2* to human gliomagenesis. *E2F2* expression was knocked down by transfecting U87MG cells with plasmids carrying a specific silencing shRNA. Upon *E2F2* silencing, *in vitro* cell proliferation was significantly reduced, as indicated by a time-course analysis of viable tumor cells. Anchorage-independent cell growth was also significantly inhibited after *E2F2* silencing, based on cell colony formation in soft agar. Subcutaneous and orthotopic xenograft models of GBM in nude mice also indicated inhibition of tumor development *in vivo,* following *E2F2* silencing. As expression of the *E2F2* gene is associated with glioblastoma stem cells and is involved in the transformation of human astrocytes, the present findings suggest that E2F2 is involved in gliomagenesis and could be explored as a potential therapeutic target in malignant gliomas.

## Introduction

Glioblastoma multiforme (GBM) is the most frequent, aggressive and lethal primary malignant tumor of the central nervous system in adults worldwide. No effective treatment for this highly aggressive and infiltrative tumor is available, and the median survival time is <16 months following initial diagnosis, with a five-year survival rate as low as 4.7% (1,2). High proportion of mitotically active cells displaying pleomorphic morphology, pseudopalisading necrosis associated with microvascular hyperplasia, and infiltrative cell growth towards the parenchyma are some of the main histopathological features of GBM. Such key features that are observed in the majority of GBM specimens can be recapitulated in orthotopic xenograft models by the intracerebral injection of patient-derived stem-like cells, which are considered to be responsible for gliomagenesis (3).

A previous genome-wide expression profiling study identified *E2F2* as a hyperexpressed gene in CD133^+^ stem-like cells isolated from fresh GBM specimens. Furthermore, the frequency and levels of *E2F2* expression correlated significantly with the malignancy of astrocytomas, being predominantly hyperexpressed in GBM (4). The E2F2 protein belongs to a large family of transcription factors regulating cell proliferation, cell division and cell differentiation. The E2F family has nine members, which have been divided into two subclasses (activators and repressors) based on their transcriptional properties and conserved structural features. E2F2 has a strong transcriptional activation domain and is able to interact with the tumor suppressor Rb (5). The Rb/E2F network regulates the expression of genes involved in cell cycle progression, DNA replication, checkpoint control, apoptosis, differentiation, DNA damage repair and development (6). Despite its well-known positive regulation of cell proliferation, the contribution of E2F2 to tumorigenesis is not so clear, since it has been reported to exert either pro-oncogenic or tumor suppression effects (7).

Since CD133^+^ GBM cells have originally been reported to have increased tumor initiating capability *in vivo* (3,8), a functional study was carried out to address the relevance of *E2F2* to the tumorigenic properties of GBM, and its value as a therapeutic target for treatment of this highly aggressive brain tumor.

## Materials and methods

### Cell culture

The human glioblastoma cell line U87MG was kindly provided by Dr Suely K. N. Marie from the Laboratory of Medical Investigation (LIM15) at the University of São Paulo (São Paulo, Brazil). Cells were grown in Dulbecco’s modified Eagle’s Medium-low glucose (DMEM-LG; Invitrogen Life Technologies, Carlsbad, CA, USA) supplemented with 2 mM L-glutamine, 10% fetal bovine serum (FBS), 100 U/ml penicillin and 100 μg/ml streptomycin (all Life Technologies, Grand Island, NY, USA), in a humidified atmosphere at 37°C with 5% CO_2_.

### Transient E2F2 silencing

U87MG cells were transfected with Sure Silencing™ shRNA plasmids (Super Array, SABiosciences, Frederick, MD, USA) designed to specifically knock down the expression of the *E2F2* gene. After 24 h without FBS for synchronization, U87MG cells were seeded in six-well plates at a density of 10^5^ cells per well and incubated for 24 h. Cells were then transfected with non-specific DNA (negative, non-specific control; NS) or shRNA silencing *E2F2* (shE2F2) (both SABiosciences, Frederick, MD, USA), using Lipofectamine™ RNAiMAX (Life Technologies) according to the manufacturer’s instructions. The total plasmid concentration in each well was 0.5 μg. Positive control cells were treated with Lipofectamine RNAiMAX, identically to the other experimental groups, but received no plasmids. Twenty-four and 72 h after transfection, the glioblastoma cells displaying neomycin resistance were selected in medium containing 500 μg/ml G418 (Life Technologies) and harvested after 96 h of culture for *in vitro* and *in vivo* experiments.

### Quantification of gene expression by quantitative polymerase chain reaction (qPCR)

Total RNA was extracted using an RNeasy^®^ mini kit (50) (Qiagen GmbH, Hilden, Germany), according to the manufacturer’s instructions, and quantified by measuring the absorbance at 260 nm (NanoDrop 2000 spectrophotometer, Thermo Fisher Scientific, Wilmington, DE, USA). The reverse transcription (RT) reaction was performed using 1 μg of total RNA with Superscript™ III Reverse Transcriptase enzyme (Life Technologies). Real-time RT-PCR was performed in a 7500 Real-time RT-PCR system (Life Technologies), by the SYBR^®^ GreenER™ incorporation method (Power SYBR Green PCR Master Mix; Life Technologies). The cycling conditions were as follows: 95°C for 15 sec, followed by 50 cycles at 60°C for 30 sec, 95°C for 1 h and 55°C for 30 sec. All primer pairs were designed in different exons using Primer3 Input version 0.4.0 (http://gmdd.shgmo.org/primer3/?seqid=47), and synthesized by Promega Corporation (Madison, WI, USA). The primer sequences were as follows: Forward, 5′-GGACAGGAATGGCCTC-3′ and reverse, 5′-GTCCTTCGAGGAGCTC-3′ for *E2F2*; and forward, 5′-GGACAGGAATGGCCTC-3′ and reverse, 5′-GTCCTTCGAGGAGCTC-3′ for *GAPDH*.

### Cell proliferation assays

U87MG cells were seeded on 96-well plates at an initial density of 5×10^4^ cells/well, and proliferation was measured 24, 48 and 72 h after plating by direct counting of viable cells in a Neubauer chamber with Trypan blue (1:1; Sigma-Aldrich, St. Louis, MO, USA). The number of viable cells was also assessed by the 3-(4, 5-dimethylthiazolyl-2)-2, 5-diphenyltetrazolium bromide (MTT) assay, adding 10 μl of MTT to the cell preparation and incubating for 2 h in a humidified atmosphere at 37°C with 5% CO_2_. Cells were lysed in 100 μl of 100% dimethylsulfoxide and absorbance was detected at 550 nm using a 96-well plate reader (iMark Microplate Absorbance Reader; Bio-Rad, Hercules, CA, USA). Each sample was run in triplicate. Anchorage-independent cell growth was also assessed by the soft agar assay. Briefly, 2 ml of 0.5% agar was added to each well of a 12-well plate. Detached U87MG cells were mixed with an agarose suspension (0.3% final concentration), and then layered onto the 0.5% agarose underlay. Culture medium was changed every three days, and the number of cell foci ≥100 mm in diameter was counted after 21 days using the EVOS^®^ XL cell imaging system (Life Technologies). Each experiment was performed in triplicate.

### In vivo tumorigenesis

#### Subcutaneous xenograft model

U87MG glioblastoma cells (10^6^ cells/mouse) were suspended in DMEM-LG, injected subcutaneously into the right flank of BALB/c nude mice (male; 4–8 weeks old) obtained from the University of São Paulo, and allowed to grow for 50 days or until the tumor reached a volume of 2,500 mm^3^ (tumor weight, 100–200 mg). Animals (n=5 per group) were monitored daily and tumors were measured with a digital caliper rule twice a week. Tumor volume was estimated using the formula: Volume = (minor diameter^2^ × major diameter)/2.

#### Orthotopic glioblastoma xenograft model

Adult BALB/c nude mice (~20 g) were anesthetized by intraperitoneal administration of ketamine (100 mg/kg)/xylazine (15 mg/kg) (both Syntec Brasil, Cotia, Brazil). Following sedation, mice were positioned in a stereotaxic frame. The scalp was sterilized with iodine and 70% ethanol and a median incision of ~1.0 cm was made. The cranial cavity was assessed by a right frontal hole using an electric mini-drill (Micromotor LB100; Beltec, Araraquara, Brazil). A total of 10^6^ U87MG cells (E2F2 knocked down and control) were suspended in 5 μl of DMEM low glucose without FBS and inoculated with a high-precision microsyringe (701RN; Hamilton Company, Reno, NV, USA) into the striatum, 0.9 mm in front of the bregma, 2.5 mm laterally to the right and 3.0 mm ventrally, at a 0.5 μl/min rate. At the end of cell injection, the needle was retained in the incision for 5 min and slowly removed to prevent the cell suspension from flowing back. The scalp was closed with 2-0 silk suture and the animals were housed under standard controlled conditions (7:00am to 7:00pm light/dark cycle; 20–22°C; 45–55% humidity) with food and water ad libitum. Histological analysis was performed 30 days post-intracranial implantation of tumor cells. All efforts were made to minimize animal suffering as proposed by the International Ethical Guideline for Biomedical Research (CIOMS/OMS, 1985). The study was approved by the ethics committee for animal research of the University of São Paulo (CEUA protocol no. 132/2011).

#### Histological analysis

Brain samples were frozen in cold isopentane solution (Sigma-Aldrich) at −25°C, and then sectioned at 20 μm on a cryostat. Coronal histological sections of the tumor xenograft and surrounding brain area were mounted on silanized microscope slides (StarFrost^®^, Knittel-Gläser, Braunschweig, Germany), and stained with hematoxylin and eosin. Microscope images were captured by an ExwaveHAD Color video digital camera (Sony Corporation, Tokyo, Japan) attached to a Nikon Eclipse E600 microscope (Nikon, Corporation, Tokyo, Japan), using the WinAVI Video Capture software (WinAVI, Eden Prairie, MN, USA).

### Statistical Analysis

All experiments were performed in triplicate and three independent experiments were performed. Data were analyzed by one way analysis of variance with Bonferroni as the post hoc test, using GraphPad Prism 3.0 (GraphPad Software, San Diego, CA, USA). P<0.05 was considered to indicate a statistically significant difference. Data are presented as the mean ± standard deviation.

## Results

### E2F2 knockdown inhibits glioblastoma cell proliferation in vitro

**Figure 1:**
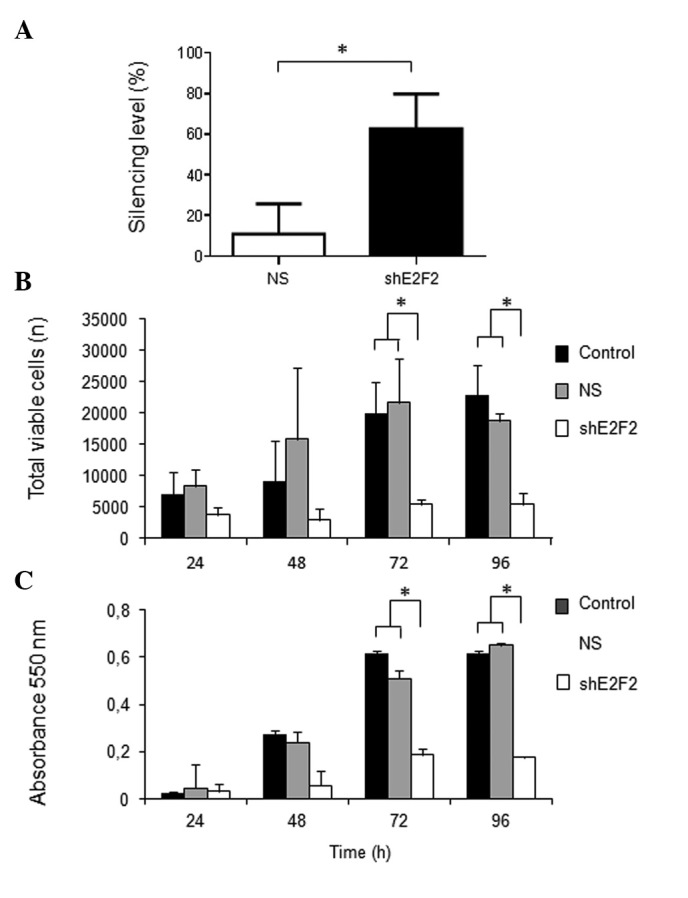
(A) Effects of *E2F2* knockdown on glioblastoma cell proliferation. *E2F2* silencing levels 96 h post-transfection of U87MG cells. Total number of viable tumor cells after 24, 48, 72 and 96 h of cell culture accessed by (B) direct cell counting or (C) 3-(4, 5-dimethylthiazolyl-2)-2, 5-diphenyltetrazolium bromide assay. Control, treated with Lipofectamine™ only; NS, non-specific control; shE2F2, *E2F2* knockdown. ^*^P<0.05.

Specific knockdown of *E2F2* expression was previously confirmed in U87MG cells, reaching a silencing level of ~60% after 96 h of transfection with shRNA. Under standard growth conditions *in vitro*, the total number of viable tumor cells was significantly lower after 48, 72 and 96 h of *E2F2* knockdown compared with that in the NS control group (P=0.0044, P=0.0007 and P=0.0035, respectively). Similar results were observed by the MTT assay, based on the activity of mitochondrial succinate dehydrogenase, which indicated significantly lower numbers of viable tumor cells after 72 h and 96 h of *E2F2* knockdown, compared with the controls (P<0.0001 for the two time points) (Fig. 1). The absorbance levels acquired from cells subjected to *E2F2* knockdown were virtually unchanged over the time course examined (24–96 h), suggesting inhibition of cell proliferation.

**Figure 2:**
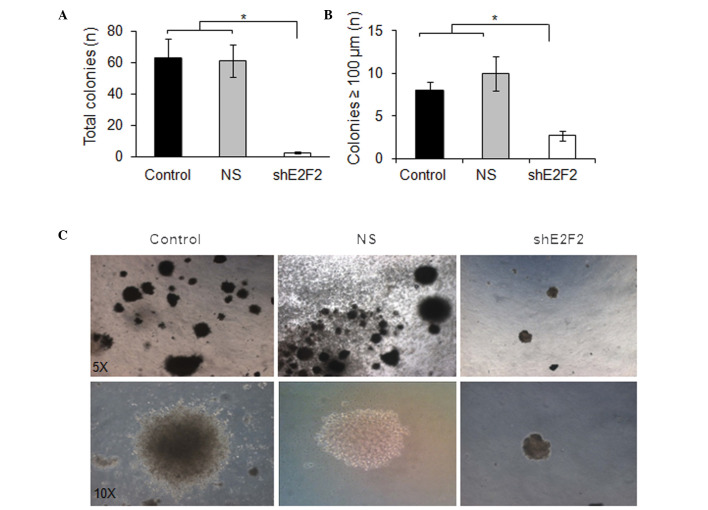
Effects of *E2F2* knockdown on glioblastoma anchorage-independent cell growth. Tumor cells were grown in soft agar medium and cell colonies counted after 21 days. (A) Total amount of colonies and (B) colonies ≥100 μm only were plotted. (C) Demonstrative images of tumor cell colonies at 5× and 10× magnification. Control, treated with Lipofectamine™ only; NS, non-specific control; shE2F2, *E2F2* knockdown. ^*^P<0.05.

Anchorage-independent cell growth is a valuable indicator of tumorigenic capability, since it is associated with neoplastic transformation and metastatic potential. In agreement with the previous cell viability experiments, the efficiency of U87MG cells to generate tumor cell colonies by anchorage-independent growth in a semi-solid medium was significantly reduced by knocking down *E2F2*. Both the total amount of colonies (≥100 μm) and the average size of the colonies were significantly lower when assaying U87MG cells subjected to *E2F2* knockdown, compared with those of the control cells (P=0.0081 and P=0.0076, respectively) (Fig. 2).

### E2F2 knockdown inhibits gliomagenesis in xenograft models

**Figure 3:**
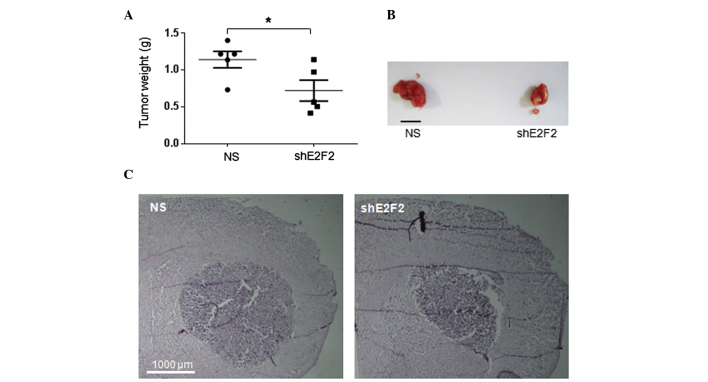
Effects of *E2F2* knockdown on tumor development. (A) Average weight of tumors derived from the subcutaneous injection of U87MG cells (10^6^ cells/mouse) and (B) representative image of resected tumors. (C) Coronal brain section of adult BALB/c nude mice bearing intracerebral tumors resulting from the stereotaxic injection of U87MG cells (106 cells/brain) into the right striatum. NS: non-specific control; shE2F2 (*E2F2* knockdown). ^*^P<0.05.

In order to test whether *E2F2* knockdown would affect *in vivo* tumorigenesis, two xenograft models of human GBM were employed. Tumors derived from the subcutaneous injection of U87MG cells in nude mice were measurable ~30 days following injection, reaching volumes usually higher than 1,000 mm^3^ in the subsequent 20 days of *in vivo* growth. In a period of 50 days post-cell injection, although transient, the E2F2 knockdown in U87MG cells inhibited tumor development. Tumors generated from U87MG cells with *E2F2* knockdown were smaller and significantly lighter than tumors resulting from control cells (P=0.04) (Fig. 3A). Mice bearing orthotopic U87MG tumors also revealed differences in brain tumor development due to *E2F2* knockdown. In agreement with the previous subcutaneous xenograft model, brain tumors derived from the stereotaxic intracerebral injection of U87MG cells with *E2F2* knockdown were somewhat smaller than tumors derived from control cells, 30 days following injection in nude mice (Fig. 3B).

## Discussion

Despite the conserved functions in cell cycle regulation, development and tissue maintenance, E2F transcription factors may affect tumorigenic processes in different ways due to the fact that each member displays individual mechanisms of action and may control the expression of other family members through a complex feedback regulation (9). The function of E2F2 is less characterized relative to other members of the E2F family, and its involvement in tumorigenesis remains a matter of debate, since evidence of both tumor suppression and pro-oncogenic activities have been reported (5).

It has been shown in mice that deficiency in E2F2 caused by gene targeting (*E2F2*^−/−^) significantly increased the population of self-reactive peripheral T cells, causing symptoms similar to severe autoimmunity. Such increment in self-reactive T cells was demonstrated to be due to increased cell proliferation rates without evidence of differential resistance to apoptosis (10). More recently, however, overexpression of *E2F2* was reported to induce p53-mediated apoptosis of mouse retina neurons lacking *Rb* and *p107*, independent of other activating E2Fs (11). In a conditional bitransgenic mouse model of Myc-induced T-cell lymphomagenesis, Opavsky *et al* (12) demonstrated that inactivation of *E2F2* (either *E2F2*^+/−^ or *E2F2*^−/−^), but not of *E2F1* or *E2F3*, significantly accelerated tumor onset and progression, indicating an haploinsufficient tumor suppressor function for *E2F2* in T cells. Similar results were obtained with MMTV-Myc transgenic mice, in which *E2F2* knockout delayed latency and reduced the incidence of Myc-driven mammary tumors (13).

By contrast, in neuroblastomas, E2F2 was shown to positively regulate *MYCN* transcription and thought to be required for full activity of *MYCN* expression in aggressive neuroblastomas usually associated with poor prognosis (14). Stable overexpression of *E2F2* in fibroblasts indeed revealed a strong oncogenic capacity for this E2F member (15). Transgenic mice have also supported a pro-oncogenic role for E2F2. In an Eμ-pp-E2F2 mouse model, overexpression of *E2F2* induced mild hyperplasia of the thymus in young mice and subsequent development of thymomas (16). Notably, overexpression of *E2F2* was predominantly found in cortical thymic epithelial cells, which are highly proliferative cells involved in T-cell development and regeneration capacity of the thymus (17). In *E2F2* knockout mice, loss of this transcription factor resulted in cell cycle arrest in hematopoietic progenitors (18) and increased DNA double-strand breaks in erythroblasts (19).

Regarding human cancers, in addition to the abovementioned study in neuroblastomas, *E2F2* has been shown to be under control of the AP-1 transcription factor in breast cancer cells, where it positively regulates cell proliferation (20). Accordingly, high *E2F2* expression was recently reported to be associated with poor survival of breast cancer patients (21). In prostate cancer cells, *E2F2* expression was reported to be inhibited by let-7a (22) and miR-31 (23) microRNAs, resulting in suppression of tumorigenesis in a nude mice ectopic xenograft model.

In GBM, *E2F2* was identified as one of the hyper-expressed genes in CD133^+^ tumor cells, compared with their counterparts, and its expression correlated with malignancy grade (4). Such subcellular population of GBM had been reported to have neural stem cells characteristics and enhanced *in vivo* tumor initiation capability (8). Studies also isolated and characterized CD133^+^ stem-like cells in different GBM cell lines, including U87MG (24), establishing a useful experimental model to study cancer stem cell biology.

The effects of *E2F2* knockdown in U87MG cells verified in the present study by *in vitro* and *in vivo* models of tumorigenesis are consistent with a pro-tumorigenic activity of E2F2 in GBM. In agreement with this notion, a recent study demonstrated that overexpression of the microRNA miR-125b inhibits proliferation of CD133^+^ GBM cells *in vitro,* by targeting *E2F2* transcripts, and that such effect on *in vitro* proliferation is rescued by overexpression of *E2F2* (25). Overall, these concordant findings suggest that E2F2 is an important transcription factor regulating the tumor-initiating capability of human GBM cells. Inhibitors of *E2F2* expression may therefore be considered as candidates for drug development to locally treat GBM, a highly malignant and devastating tumor of the central nervous system.

## References

[1] Karak AK, Singh R, Tandon PN, Sarkar C (2000). A comparative survival evaluation and assessment of interclassification concordance in adult supratentorial astrocytic tumors. Pathol Oncol Res.

[2] Dolecek TA, Propp JM, Stroup NE, Kruchko C (2012). CBTRUS statistical report: primary brain and central nervous system tumors diagnosed in the United States in 2005–2009. Neuro Oncol.

[3] Dimova DK, Dyson NJ (2005). The E2F transcriptional network: old acquaintances with new faces. Oncogene.

[4] Bracken AP, Ciro M, Cocito A, Helin K (2004). E2F target genes: unraveling the biology. Trends Biochem Sci.

[5] DeGregori J (2002). The genetics of the E2F family of transcription factors: shared functions and unique roles. Biochim Biophys Acta.

[6] Okamoto OK, Oba-Shinjo SM, Lopes L, Nagahashi Marie SK (2007). Expression of HOXC9 and E2F2 are up-regulated in CD133(+) cells isolated from human astrocytomas and associate with transformation of human astrocytes. Biochim Biophys Acta.

[7] Galli R, Binda E, Orfanelli U (2004). Isolation and characterization of tumorigenic, stem-like neural precursors from human glioblastoma. Cancer Res.

[8] Singh SK, Hawkins C, Clarke ID (2004). Identification of human brain tumour initiating cells. Nature.

[9] Chen HZ, Tsai SY, Leone G (2009). Emerging roles of E2Fs in cancer: an exit from cell cycle control. Nat Rev Cancer.

[10] Murga M, Fernández-Capetillo O, Field SJ (2001). Mutation of E2F2 in mice causes enhanced T lymphocyte proliferation, leading to the development of autoimmunity. Immunity.

[11] Chen D, Chen Y, Forrest D, Bremner R (2013). E2f2 induces cone photoreceptor apoptosis independent of E2f1 and E2f3. Cell Death Differ.

[12] Opavsky R, Tsai SY, Guimond M (2007). Specific tumor suppressor function for E2F2 in Myc-induced T cell lymphomagenesis. Proc Natl Acad Sci USA.

[13] Fujiwara K, Yuwanita I, Hollern DP, Andrechek ER (2011). Prediction and genetic demonstration of a role for activator E2Fs in Myc-induced tumors. Cancer Res.

[14] Strieder V, Lutz W (2003). E2F proteins regulate MYCN expression in neuroblastomas. J Biol Chem.

[15] Chen C, Wells AD (2007). Comparative analysis of E2F family member oncogenic activity. PLoS One.

[16] Scheijen B, Bronk M, van der Meer T, De Jong D, Bernards R (2004). High incidence of thymic epithelial tumors in E2F2 transgenic mice. J Biol Chem.

[17] Rode I, Boehm T (2012). Regenerative capacity of adult cortical thymic epithelial cells. Proc Natl Acad Sci USA.

[18] Li FX, Zhu JW, Hogan CJ, DeGregori J (2003). Defective gene expression, S phase progression, and maturation during hematopoiesis in E2F1/E2F2 mutant mice. Mol Cell Biol.

[19] Dirlam A, Spike BT, Macleod KF (2007). Deregulated E2f-2 underlies cell cycle and maturation defects in retinoblastoma null erythroblasts. Mol Cell Biol.

[20] Shen Q, Uray IP, Li Y, Krisko TI, Strecker TE, Kim HT, Brown PH (2008). The AP-1 transcription factor regulates breast cancer cell growth via cyclins and E2F factors. Oncogene.

[21] Nguyen-Vu T, Vedin LL, Liu K (2013). Liver X receptor ligands disrupt breast cancer cell proliferation through an E2F-mediated mechanism. Breast Cancer Res.

[22] Dong Q, Meng P, Wang T (2010). MicroRNA let-7a inhibits proliferation of human prostate cancer cells in vitro and in vivo by targeting E2F2 and CCND2. PLoS One.

[23] Lin PC, Chiu YL, Banerjee S (2013). Epigenetic repression of miR-31 disrupts androgen receptor homeostasis and contributes to prostate cancer progression. Cancer Res.

[24] Yu SC, Ping YF, Yi L (2008). Isolation and characterization of cancer stem cells from a human glioblastoma cell line U87. Cancer Letters.

[25] Wu N, Xiao L, Zhao X (2012). miR-125b regulates the proliferation of glioblastoma stem cells by targeting E2F2. FEBS Lett.

